# Conserved community structure and simultaneous divergence events in the fig wasps associated with *Ficus benjamina* in Australia and China

**DOI:** 10.1186/s12898-018-0167-y

**Published:** 2018-04-03

**Authors:** Clive T. Darwell, Simon T. Segar, James M. Cook

**Affiliations:** 1 School of Biological Sciences, University of Reading, Reading, RG6 6AS UK; 2 Okinawa Institute of Science and Technology, 1919-1 Tancha, Onna-son, Okinawa 904-0495 Japan; 3 Biology Centre of the Czech Academy of Sciences, Institute of Entomology and Faculty of Science, University of South Bohemia, Branisovska 31, 370 05 Ceske Budejovice, Czech Republic; 4 Hawkesbury Institute for the Environment, Western Sydney University, Locked Bag 1797, Penrith, NSW 2751 Australia

**Keywords:** Barcoding, Chalcidoidea, Community composition, Biodiversity, *Ficus*, Galler, Parasitoid, Wasp

## Abstract

**Background:**

Localised patterns of species diversity can be influenced by many factors, including regional species pools, biogeographic features and interspecific interactions. Despite recognition of these issues, we still know surprisingly little about how invertebrate biodiversity is structured across geographic scales. In particular, there have been few studies of how insect communities vary geographically while using the same plant host. We compared the composition (species, genera) and functional structure (guilds) of the chalcid wasp communities associated with the widespread fig tree, *Ficus benjamina*, towards the northern (Hainan province, China) and southern (Queensland, Australia) edges of its natural range. Sequence data were generated for nuclear and mtDNA markers and used to delimit species, and Bayesian divergence analyses were used to test patterns of community cohesion through evolutionary time.

**Results:**

Both communities host at least 14 fig wasp species, but no species are shared across continents. Community composition is similar at the genus level, with six genera shared although some differ in species diversity between China and Australia; a further three genera occur in only China or Australia. Community functional structure remains very similar in terms of numbers of species in each ecological guild despite community composition differing a little (genera) or a lot (species), depending on taxonomic level. Bayesian clustering analyses favour a single community divergence event across continents over multiple events for different ecological guilds. Molecular dating estimates of lineage splits between nearest inter-continental species pairs are broadly consistent with a scenario of synchronous community divergence from a shared “ancestral community”.

**Conclusions:**

Fig wasp community structure and genus-level composition are largely conserved in a wide geographic comparison between China and Australia. Moreover, dating analyses suggest that the functional community structure has remained stable for long periods during historic range expansions. This suggests that ecological interactions between species may play a persistent role in shaping these communities, in contrast to findings in some comparable temperate systems.

**Electronic supplementary material:**

The online version of this article (10.1186/s12898-018-0167-y) contains supplementary material, which is available to authorized users.

## Background

Recent decades have heralded the realisation that to understand how species diversity is generated, maintained and distributed we must investigate the evolutionary histories of organisms in conjunction with regional patterns and processes [1–3]. Moreover, as diversity is composed of species whose evolutionary viability depends partly on species interactions, it is important to investigate patterns of biodiversity in a community context [4, 5]. In geographically wide-ranging communities, there may be species turnover along environmental and geographical gradients, leading to high β-diversity (the degree of turnover in species composition across sites), even if measures of local α-diversity remains the same [6]. If ecological processes are the primary force governing species’ occurrences, one would predict convergence in community structure at a regional scale given similar local abiotic and biotic conditions. Alternatively, spatial turnover of species may facilitate divergence in community composition and structure between environmentally and geographically distinct regions [7, 8], if species are lost or gained.

While considerations of community structure often assume that niche space is filled, geographic variation in local species diversity may indicate vacant niches in some populations [9–11]. Communities utilising the same resources in geographically disparate regions may be taxonomically (and even functionally) dissimilar [11], suggesting that community composition and structure may depend more strongly on stochastic recruitment processes, the regional species pool, phylogenetic constraints, or intrinsic differences in the key resources upon which communities are founded, rather than being driven by interspecific interactions.

One approach to better identify underlying processes, both within and between trophic levels, and determine drivers of community assembly at wide geographic scales, is to compare the divergence histories of interacting species [12]. Communities in which species have dispersed at different times from source populations have likely experienced intermittent interspecific interactions. Such a history reduces potential for coevolutionary dynamics to shape community assembly and increases the opportunities for colonisation of certain niches by other species. This may facilitate ecological dominance for early colonisers that can monopolise resources and impede species at higher trophic levels that cannot invade new populations until their target resources arrive.

Alternatively, continuous associations among species are consistent with strong coevolutionary interactions leading to more specialised life-history strategies, a potential concomitant increase in diversity [13], and a reduced capacity for species-pool recruitment due to stabilised ecological interactions. In one well-studied system—communities of Palearctic oak gall wasps and their parasitoids—population histories are dominated by Pleistocene glacial maxima that restricted species to southern refugia [12]. Subsequent diversification among these species occurred in western Asia and was followed by westward pulses of range expansion, with gall-making herbivores preceding their parasitoid predators and thus “enjoying” periods of enemy-free space. It is unknown whether such patterns are common in tropical systems, which are unlikely to have been so strongly affected by glacial refugia.

Herbivorous insects and their parasitoid enemies comprise a large proportion of terrestrial biodiversity. As such they are of profound ecological importance and engage in a vast repertoire of trophic interactions [14–17]. Nevertheless, relatively little is known about the diversity, distribution and biology of many such insect groups, especially in tropical regions, and there have been few studies comparing herbivore and parasitoid assemblages on the same host plant between geographical regions [18]. Many insect herbivores, and the majority of all parasitoids, belong to the insect order Hymenoptera [19] and a number of studies have focused on the multitrophic insect communities associated with the fig-pollinator mutualism [e.g. 20–26].

*Ficus* is a ubiquitous tropical and sub-tropical resource, comprising around 750 species that provide year-round fruit resources for diverse animals. Fig-wasp community structure is complex, typically involving coexistence of five functional groups [21] or ecological guilds. Small herbivorous wasp species may be pollinators (guild 1) or non-pollinators (guild 2) with both groups galling fig flowers. These small galler guilds are exploited by (guild 3) kleptoparasites/inquilines (that usurp these galls) or parasitoids. Large wasp species, in turn, may be gallers (guild 4) of fig wall tissue or flowers, or parasitoids (guild 5) of large gallers. While several fig wasp communities have been studied, there have been few comparisons of entire fig wasp communities in different geographic areas [26–28] and none using molecular tools to investigate diversity and divergence histories (but see [29] for work focussed on particular fig wasp guilds).

Here, we focus on the wasp community of the tropical fig species, *Ficus benjamina* (Conosycea, Moraceae), which is widespread and common from India to China at the northern limits of its range, and through Southeast Asia to northern regions of Australia at its southern limits. It thus occurs in both the Oriental and Australasian regions. *Ficus* section Conosycea is thought to have originated in India and radiated across Asia around 35 Mya [30]. After this, it may have ‘rafted’ across the Wallace line on a fragment of Borneo, as is hypothesised for many Asian plants. We compare the morphological and molecular community description of *F. benjamina* fig wasps in Hainan province, China [22] with our own data for the Australian *F. benjamina* fig wasp community in Queensland (Australia) to investigate how the species composition and trophic structure of the community varies across its wide geographic range.

We employ molecular species delimitation methods to ascertain whether species from China are also present in Australia and to ascribe genera into taxonomically associated functional groupings that we refer to as ecological guilds. Given the wide geographic separation of the two communities, we predicted few shared species. However, as the two communities occupy similar (northern and southern) latitudes, we predicted that they should display convergent community composition at higher taxonomic levels (genera, subfamilies). Further, we use molecular dating methods to establish the degree of community cohesion across evolutionary time. As previous work on temperate oak gall wasp communities has shown evidence of temporal tracking of parasitoid species following their galling hosts during range expansion [12], we expected to see similar patterns among these fig wasp communities.

## Methods

### Study system and sampling

**Fig. 1:**
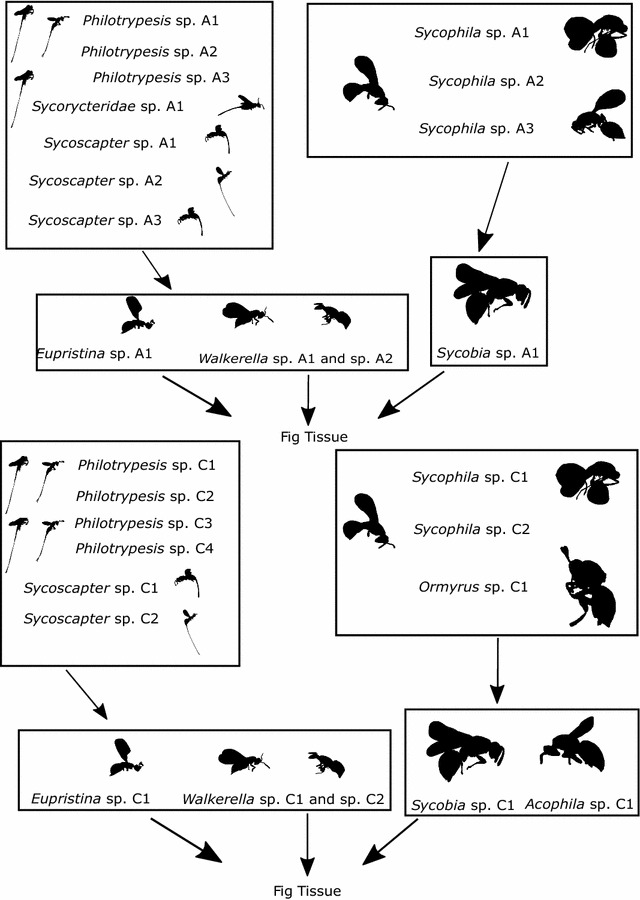
A graphical summary of the proposed trophic interactions between the wasps associated with *F. benjamina*. Summaries of network structure are given for both Australian (AUS), above, and Chinese (CN), below, networks. Genus level representative silhouettes of wasps are given for size comparison and visualisation purposes only. Alpha-numeric species designations are assigned according to region and genus; e.g., Australian *Walkerella* species 1 is assigned ‘*Walkerella* A1’

Xiao et al. [22] identified seven genera of chalcid fig wasps from their sampled community in Hainan province, China (19.2175°N, 109.8127°E). *Ficus benjamina* is pollinated by *Eupristina koningsbergeri*, which, along with the externally ovipositing *Walkerella* spp. (Pteromalidae, Otitisellinae), comprise the small galling species in this community. Attacking these are the small parasitoid genera *Philotrypesis* and *Sycoscapter* (Pteromalidae, Sycoryctinae). Among the large wasps, *Sycobia* and *Acophila* (both Epichrysomallinae) are large gallers, whilst their parasitoid enemies are from the genus *Sycophila* (Eurytomidae). Additionally, Xiao et al. [22] identified two individuals of *Ormyrus* (Ormyridae), a rare large species whose life habit is unknown. Figure 1 shows the simplified community structure of *F. benjamina* fig wasps according to our current understanding of their ecological guilds distinguished according to trophic role and adult wasp body size [21].

The Australian wasp community from *F. benjamina* figs was sampled periodically in northern Queensland, from Lake Eacham (17.2848°S, 145.6259°E; 1998/99), Yungaburra (17.2705°S, 145.5829°E; 2007), Cairns (16.9127°S, 145.7709°E; 1995, 1998/99, 2003, 2004, 2007 and 2008) and Townsville (19.2575°S, 146.8178°E; 2008). Near-ripe figs were collected from trees and placed into plastic hatching pots with meshed lids that allow air-flow, but prevent overheating and wasp escape. After 48 h each fig and any emerged wasps were placed into 70% ethanol. Alternatively, figs were placed directly into alcohol and wasps were dissected out of their galls at a later date. Wasps were easily identified to genus using the key of Bouček [31] and cross-checking with the images of Xiao et al. [22]. Species level designations were then made using molecular data, since the majority of fig wasp species remain undescribed.

We sequenced the following numbers of individuals from each constituent wasp (sub)family: Agaonidae: *Eupristina* (9); Sycoryctinae: *Philotrypesis* (50); *Sycoscapter* (18); *Sycorycteridea* (6); Epichrysomallinae: *Sycobia* (9); Eurytomidae: *Sycophila* (14); Otitesellinae: *Walkerella* (24). The genetic sampling effort reflects our perception of the morphological diversity we observed within each genus. Individuals for sequencing were chosen to represent observed colour and size variation. For genera where sample numbers are low (i.e. < 10 individuals), sample sizes represent the maximum number of individuals available to us, i.e. without sequencing multiple non-independent monomorphic individuals from the same figs. Although it would have been better to have identical sampling schemes across communities, there is no information in Xiao et al. [22] regarding sampling effort. However, as we have sampled a similar number of individuals and genera, it is likely that the sampling effort employed in both studies is similar.

For molecular species comparisons, we used the corresponding sequence data for wasps from the study of *F. benjamina* in Hainan province, China [22], downloaded from Genbank (Accession Numbers: FJ438013–FJ438369). The Chinese dataset comprises 70 COI and ITS2 sequences with the following sample sizes by genus: *Eupristina koningsbergeri*-6; *Sycophila*-9; *Philotrypesis*-17; *Sycoscapter*-9; *Walkerella*-20; *Acophila*-6 (Epichrysomallinae); *Sycobia*-7; and *Ormyrus*-2 (Ormyridae).

### Molecular methods

A Chelex method [32] was used to extract DNA from Australian samples and a mitochondrial (cytochrome c oxidase subunit I-COI; primers CI-J-1751 and CI-N-2191 [33]) and a nuclear (Internal Transcribed Spacer region 2-ITS2 [34]) marker were amplified. This region of COI is shorter than the standard barcoding region, as alternate primers have been designed to avoid amplification of nuclear pseudo-genes [see 22]. Amplification of COI was carried out using a Techne Touchgene gradient machine with 5 min at 94 °C, 30 cycles of 30 s at 94 °C, 45 s at 50 °C, 60 s at 72 °C then 10 min at 72 °C. Amplification of ITS2 was with 5 min at 94 °C, 35 cycles of 30 s at 94 °C, 40 s at 55 °C, 40 s at 72 °C then 10 min at 72 °C. Subsequent purification and sequencing reactions were conducted by Beckman Coulter. Purification was performed using ethanol precipitation and sequencing by BigDyeTM terminator cycling conditions and a 3730xl DNA analyser.

### Sequence alignment, phylogenetic analysis and species delimitation

New sequences were checked using the chromatogram visualisation software Finch TV Version 1.4.0 and were edited and aligned using BioEdit [35]. Final adjustments were made by eye. Bayesian methods were employed to construct phylogenies for each marker using MrBayes [36]. The best model of nucleotide substitution for each gene was determined using likelihood ratio tests with MrModeltest in PAUP* [37]. For both markers the general time reversible (GTR + I + R) model of evolution was chosen.

Species richness was calculated using the barcoding criteria of monophyletic lineages of individuals (here for both markers) with at least one other conspecific within a commonly used cut-off threshold of 3% K2P (Kimura-2-parameter measure of pairwise genetic distance [38]) pairwise difference in the COI marker [39], or the 95% confidence interval of all intraspecific distances [40]. COI K2P distances were calculated [in Mesquite; 41] for congeneric sister clades of wasps from different continents. We then further tested these species hypotheses on COI sequences using the jMOTU software package [42] that is designed to cluster sequences according to a range of cut-off values—an inflection point in jMOTU outputs purportedly shows the barcoding gap.

### Historical divergence

**Fig. 2:**
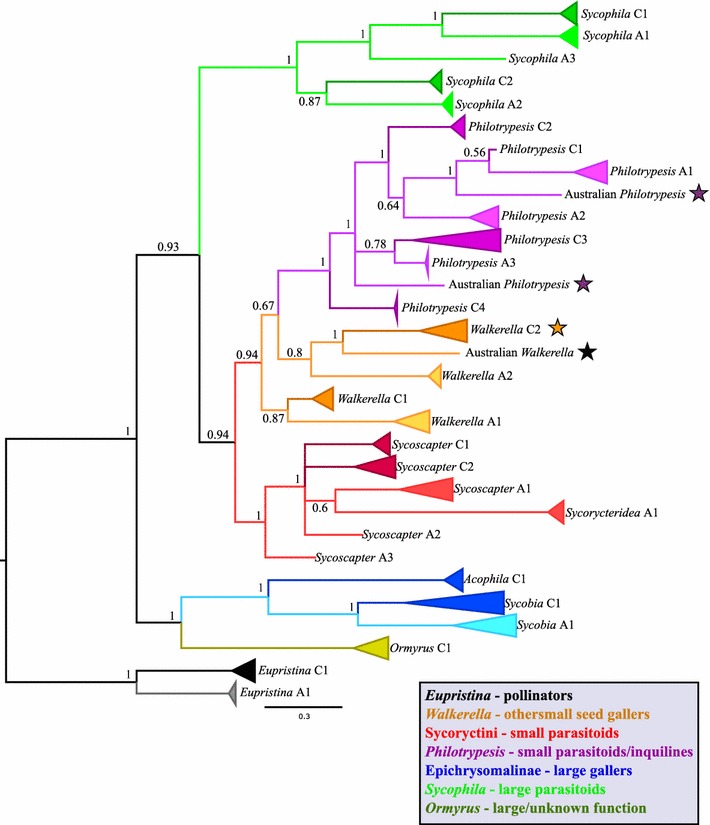
Collapsed consensus Bayesian topology of the mtDNA COI region for 158 chalcid fig-wasps from *F. benjamina* from China and Australia. All posterior node probabilities > 0.95 except where noted. Clade and terminal branch colours represent taxonomic groupings of taxa correlating with ecological function (see legend). Within individual groupings, darker colours represent Chinese samples. Stars denote notable features discussed in the results section (i.e. taxa without alpha-numeric species designations)

**Fig. 3:**
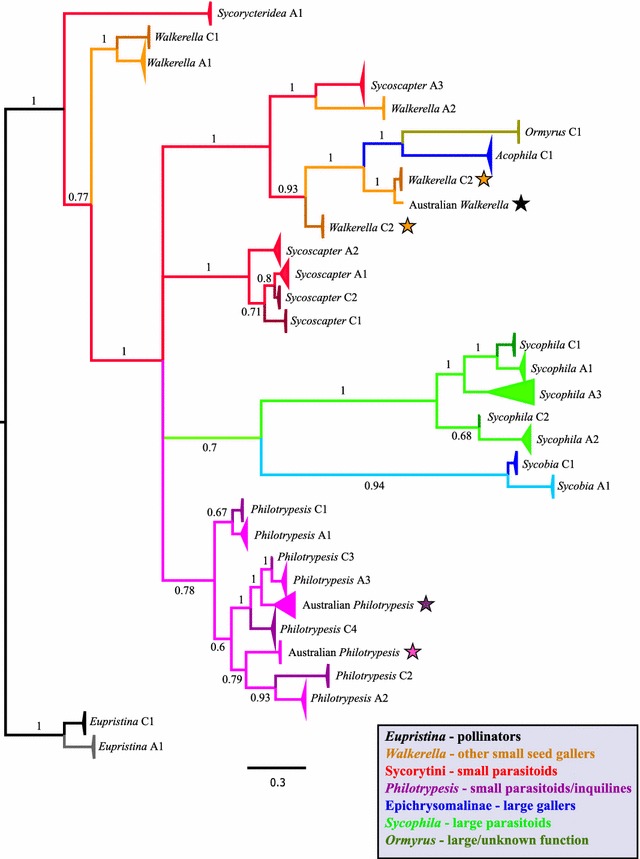
Collapsed consensus Bayesian topology of the nuclear ITS2 region for 205 chalcid fig-wasps from *F. benjamina* from China and Australia. All posterior node probabilities > 0.95 except where noted. Clade and terminal branch colours represent taxonomic groupings of taxa correlating with ecological function (see legend). Within individual groupings, darker colours represent Chinese samples. Stars denote notable features discussed in the results section

To test if intercontinental divergences were clustered by ecological guild we used a hierarchical approximate Bayesian computation (hABC) coalescent approach that allows for species-specific demographic variation and mutation rate heterogeneity [PyMsBayes; 43] on our COI data. To test different hypotheses of clustered species divergences we used a subset of eight species pairs chosen according to the following criteria: each species (China and Australia) had at least two representative COI sequences, and clear intercontinental splits between clades (Figs. 2, 3) could be identified in both genes for the species pair. Thus, we conducted pairwise comparisons on: *Eupristina* (species C1 & A1), *Philotrypesis* (species C3 & A3), *Sycoscapter* (species C1 & A1), *Walkerella* (species C1 & A1), *Sycobia* (species C1 & A1) and *Sycophila* (species C2 & A2), for our analyses. To guard against sensitivity of results to prior selection decisions we selected priors according to three separate divergence scenarios (two, four and six divergences) following instructions from the PyMsBayes manual (http://joaks1.github.io/PyMsBayes/; Accessed May 2016). We set a diffuse concentration shape parameter of 2 and set theta parameters according to the molecular clock rate of Lin et al. [1.9%; 44]. All other settings were left as default.

We evaluated several models based on differential divergences according to combinations of guilds to test individual hypotheses that certain ecological guilds diversified earlier or later than others. The evaluated models were: all species diverged simultaneously, galling species diverged before parasitoids, small species diverged before large species, all small species diverged simultaneously, small gallers diverged before small parasitoids, pollinators and small parasitoids diverged simultaneously, pollinators diverged before small parasitoids, all large species diverged simultaneously, and large gallers diverged before large parasitoids. We then used Bayes factors both to evaluate support for each individual model and then for direct comparisons between specific scenarios using the criteria of Raftery [45] to infer preferential support between models.

### Divergence dating and community structure

Split dates between congeneric species were estimated from COI data using Bayesian dating analyses in BEAST v.1.7.1 [46]. As mitochondrial DNA (mtDNA) evolves quickly and relationships among deeper nodes lose signal (e.g. within a multi-family phylogeny), we ran individual analyses on the following genera: *Eupristina*, *Walkerella*, *Philotrypesis*, *Sycobia* and *Sycophila*, as well as on the Sycoryctini tribe (*Sycoscapter* and *Sycorycteridea*). As no fossil calibration points are available, a molecular clock, estimated for Asian fig wasps (1.9% pairwise divergence per million years; [44, 47]), was imposed. Bayes factors (BF) were calculated using Raftery’s [45] logarithmic scale (2log_*e*_(BF)) to detect the most suitable models of substitution, molecular clock and speciation (where values of 2–5 on this scale are considered positive evidence, > 5 is strong evidence, and > 10 is very strong evidence). Two independent runs of 120 million iterations were run for each species separately. Genealogies and model parameters were sampled every 6000 iterations and 95% highest posterior densities (HPD) were employed as credibility intervals. The first 2000 trees of each run were discarded as the “burn-in” period.

## Results

### Sequencing results

For the Australian wasps, we obtained sequences from 117 individuals for ITS2 and 83 for COI. Several individuals could not be amplified successfully for one marker despite successful amplification of the other. The COI sequences were standardised to a 394 bp fragment alignment, while ITS2 sequences varied between 328–562 bp according to genus: *Sycobia*—557–562 bp; *Philotrypesis*—341–360 bp; *Sycophila*—328–456 bp; *Walkerella*—346–348 bp; *Sycorycteridea*—306–314 bp; *Sycoscapter*—365–405 bp, with sequence length variation attributable to indel events.

### Species delimitation

Identification of congruent monophyletic lineages between Bayesian phylogenies constructed from COI and ITS2 markers suggests that wasp communities in China and Australia each have 14 species (Table 1; Figs. 2, 3; Additional file 2). Species assignations were congruent between molecular markers except for two cases (Australian *Philotrypesis* species 4 and Chinese *Walkerella benjamini*—dark purple stars and orange stars respectively in Figs. 2, 3). Additionally, two instances among Australian samples warrant consideration as possible additional species: in *Philotrypesis*, a coherent clade of three individuals is apparent in the ITS2 phylogeny (pink star: Fig. 3) that did not yield PCR products for COI; and within *Walkerella*, a singleton wasp appears as a distinct clade for both genes (black star). Most identified species meet the barcoding criteria employed for COI sequence data. However, a few individuals among Chinese *Sycobia* and Chinese *Philotrypesis* species 3 do not have a putative conspecific falling within the thresholds limits (Table 2). Nevertheless, for all these individuals, their most closely matched individual resides within their continental monophyletic clade. For each community, intrageneric pairwise COI K2P distances are generally similar between continents (Table 1). However, notable exceptions include Australian *Sycoscapter* COI distances (~ 3%), which are much lower than for their Chinese counterparts (~ 14%), and Chinese *Sycobia* (~ 11%), that have greater divergences than the Australian species (~ 4%). Nearly all of these species hypotheses are supported by jMOTU analyses conducted on COI data (Additional file 1: Figure S1). However, the results for *Sycobia* (Additional file 1: Figure S1e) are ambiguous due to the Chinese samples, which display large pairwise genetic distances and form polytomies in the COI phylogeny. Nevertheless, ITS2 (Additional file 2: Figures S2, S3) data suggest that they are a cohesive species.

**Table 1:** Fig wasp communities found in Chinese and Australian populations of *F. benjamina*

Genus	Ecological guild	China		Australia	
		No. of species	Intrageneric K2P (%)	No. of species	Intrageneric K2P (%)
*Eupristina*	Small galler	1	< 1.2	1	< 0.5
*Philotrypesis*	Small parasitoid	4	< 19	3^a^	< 13
*Sycoscapter*	Small parasitoid	2	< 14	3	< 3
*Sycorycteridea*	Small parasitoid	–	–	1	< 2
*Walkerella*	Small galler	2	< 17	2^a^	< 14
*Sycobia*	Large galler	1	< 11	1	< 4
*Sycophila*	Large parasitoid	2	< 16	3	< 18
*Acophila*	Large galler	1	< 2	–	–
*Ormyrus*	Large parasitoid	1	–	–	–
Total		14		14^a^	

Intrageneric K2P (%) indicates maximum range of pairwise Kimura 2-parameter distances for mtDNA COI across congeneric species within communities

^a^Possible extra species

**Table 2:** Kimura 2-parameter (K2P) distances between congeneric taxa and their nearest alternate continental neighbours

	*Sycobia*	*Sycophila*	*Sycophila*	*Walkerella*	*Walkerella*	*Philotrypesis*	*Philotrypesis*
		Chi sp1–Aus sp1	Chi sp2–Aus sp2	Chi sp1–Aus sp1	Chi sp2–Aus sp2	Aus sp3–Chi sp3	Chi sp1–Aus sp1
Intraspecific K2P (%)	0–11	0–2	0–1	0–5.1	0–5	0–5.8	0–2.7
Interspecific K2P (%)	6.5–14.4	12–13	12–13	5.9–12	11–14	4.2–6.1	6–8

### Comparative community composition

Most genera were found in both continents, but *Ormyrus* and *Acophila* were present only in China, and *Sycorycteridea* only in Australia. The Australian community also harbours more species among the genera *Sycoscapter* and *Sycophila*. However, both the Chinese and the Australian communities possess a full complement of both small and large gallers and parasitoids and therefore display the full range of fig wasp guilds. None of the 28 (or more) species found in China and Australia are shared between the two continents (Figs. 2, 3). However, for both genes there are seven instances in which intercontinental species pairs are more closely related to each other than they are to a local congener (e.g., in *Walkerella*, *Philotrypesis* and *Sycophila*; Table 2 records pairwise genetic distances for these cross-continental phylogenetic sister species pairs).

### Historical divergence

Analyses of community divergence in PyMsBayes did not support temporal clustering of distinct divergence events according to ecological guilds (Table 3). In all scenarios, a model of a single divergence event was favoured over models of multiple divergences with 7/12 tests resulting in substantial support (i.e. 2log_e_(BF) > 2; [45]) for the single divergence event. The two, four and six divergence scenarios all gave a similar estimate of around 5.2 ± 1.2 Ma for the single diversification event.

**Table 3:** PyMsBayes evaluation of temporal clustering of divergence events according to various combinations of ecological guilds of intercontinental species pairs

Divergence model	Two divergence events			Four divergence events			Six divergence events		
	Posterior probability	Prior probability	Bayes factor	Posterior probability	Prior probability	Bayes factor	Posterior probability	Prior probability	Bayes factor
*All taxa*									
All synchronised	0.696	0.422	3.142**	0.382	0.081	6.978**	0.164	0.011	18.381**
Small > large	0	0.00072	0	0	0.00048	0	0	→0	→0
Gallers > parasitoids	0	0.00024	0	0.002	→0	→0	0.001	→0	→0
*Small wasps*									
All synchronised	0.719	0.475	*2.828*	0.411	0.115	*5.395*	0.185	0.021	10.400*
Gallers > parasitoids	0.011	0.008	1.437	0.013	0.005	2.875	0.004	0.0029	1.390
*Pollinators and small parasitoids*									
Pollinators = parasitoids	0.733	0.531	*2.420*	0.424	0.157	*3.945*	0.205	0.030	*8.474*
Pollinators > parasitoids	0.043	0.0317	1.372	0.062	0.0312	2.052	0.032	0.011	3.097
*Large wasps*									
All synchronised	0.814	0.582	3.138*	0.554	0.211	4.646*	0.303	0.0576	7.113*
Gallers > parasitoids	0.02	0.051	0.381	0.055	0.071	0.767	0.089	0.0466	2.000

Data are shown for three separate runs with priors set according to two, four and six divergence events. Bayes factors (BF) in italics represent favoured model. Asterisk indicates positive evidence (i.e. 2log_e_(BF) is between 2–5) for favoured model [45]. Two asterisks indicate very strong evidence (NB where posterior probability = 0, support calculation effectively becomes 2log_e_(∞)). Pairwise comparison were made between all scenarios under each sub-heading (in italics). →0 indicates tending to zero

### Divergence dating

**Fig. 4:**
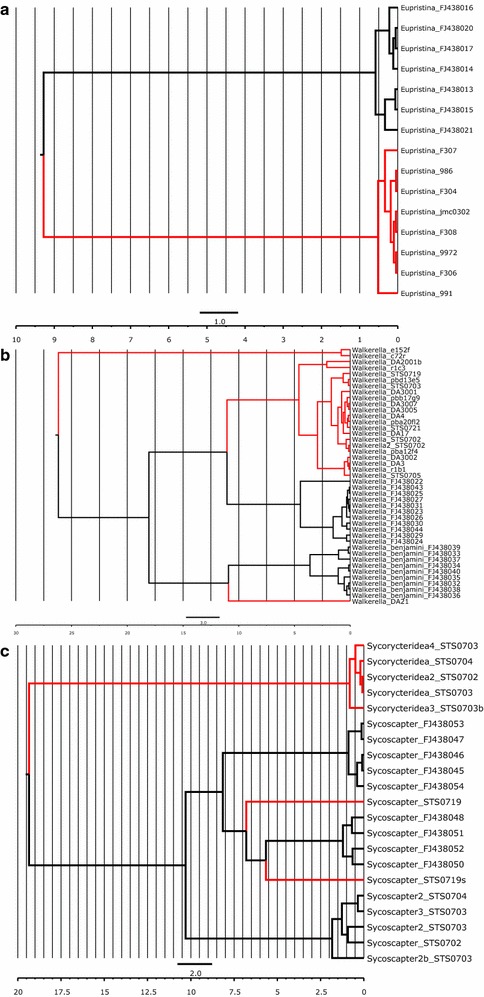
Bayesian estimation of intrageneric divergence times for fig wasp species in *F. benjamina* (**a**) *Eupristina*; **b** *Walkerella*; **c** tribe Sycoryctini. Divergence between inter-continental species occurred ca. 9–10 Mya in *Eupristina;* ca. 11–25 Mya in *Walkerella*; ca. 6–11 Mya in *Sycoscapter* (ca. 19 Mya in *Sycorycteridea*); ca. 4–16.5 Mya in *Philotrypesis*; ca. 15 Mya in *Sycobia*; 8–14 Mya in *Sycophila*. Australian lineages in red

**Fig. 5:**
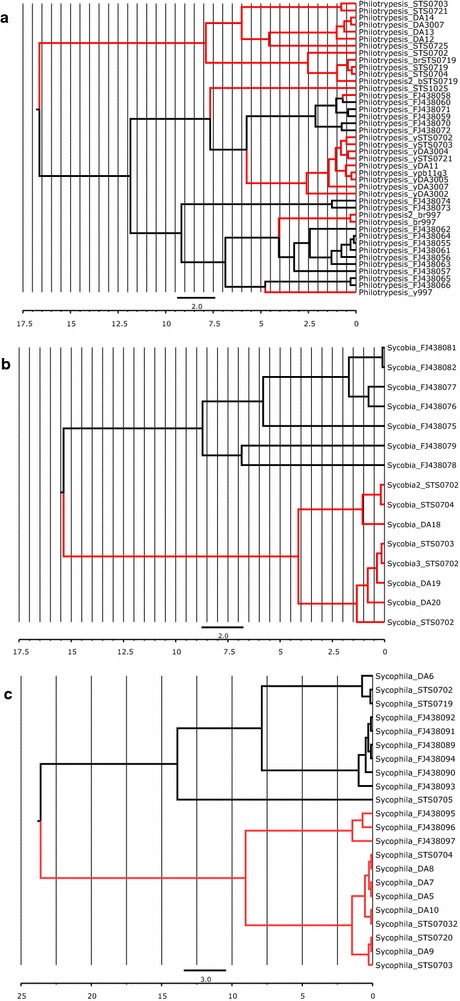
Bayesian estimation of intrageneric divergence times for fig wasp species in **a** *Philotrypesis*; **b** *Sycobia*; **c** *Sycophila*. Divergence between inter-continental species occurred ca. 9–10 Mya in *Eupristina;* ca. 11–25 Mya in *Walkerella*; ca. 6–11 Mya in *Sycoscapter* (ca. 19 Mya in *Sycorycteridea*); ca. 4–16.5 Mya in *Philotrypesis*; ca. 15 Mya in *Sycobia*; 8–14 Mya in *Sycophila*. Australian lineages in red

For molecular dating in BEAST, Bayes Factors indicated that sequence alignments for all taxa should be partitioned into three separate codons with several distinct models for sequence evolution, molecular clock and tree prior (Additional file 1: Table S1). Bayesian estimates of intrageneric divergence times between reciprocal Australian–Chinese clades suggest that these communities diverged ca. 4–16.5 Ma (Figs. 4, 5). The deeper species splits evident in *Walkerella* (17.5 and 25 Ma) are not between obvious reciprocal Australian–Chinese counterpart species pairs and may have occurred external to the *F. benjamina* microcosm (Figs. 4b, 5). In general, galling species appear to have diversified before the parasitoids, supporting the temporal tracking hypothesis. However, the most ancient split of 16.5 Ma is not in gallers but in the kleptoparasite genus *Philotrypesis*, and occurred before the split for the obligate pollinator *Eupristina* (9 Ma; but see “Discussion”). Notably, the large 95% HPD error bars mean that the divergence time estimates should be treated with some caution.

## Discussion

There is a dearth of knowledge about how a given community of interacting insect species varies across wide geographic scales. A key question is whether high beta diversity typically results in geographic differences in community functional diversity or degree of ecological saturation. Moreover, to understand the coevolutionary dynamics underpinning widespread communities we must compare the demographic histories of interacting species to determine whether or not lineages have diverged simultaneously across regions. If so, they are likely to have experienced persistent stable interspecific interactions and coevolutionary partners. Here we examined the predominantly host-specific wasp communities associated with an individual fig species, *F. benjamina*, to investigate these patterns in tropical regions. Our results show that genus-level taxonomic composition and species richness are largely conserved between China (Oriental region) and Australia (Australasian region), which suggests conserved functional diversity. Moreover, in contrast to data from comparable temperate insect communities [12], interacting lineages appear to have generally diversified simultaneously across regions, which implies greater ecological stability and a reduced role for localised asymmetries in functional dynamics.

### Species composition

Molecular species delimitation methods employing both mitochondrial and nuclear markers indicate 14 well-defined species in each of the Chinese and Australian *F. benjamina* communities. Whilst the various delimitation criteria gave predominantly congruent species hypotheses, it should be recognised that sole reliance on molecular methods invokes some level of subjective assessment. In our study this is most evident when considering the observed variance in intra- and interspecific K2P distances (Table 2), which highlight the arbitrary nature of employing rigid threshold cut-off values in COI barcoding [e.g. 48]. Although most sampled genera are found in both regions, one or more are absent from either region (i.e. *Ormyrus*, *Acophila* and *Sycorycteridea*). Although we did not record *Ormyrus* from *F. benjamina* in Australia, it may be present but rare, as in China where Xiao et al. [22] only reported two individuals. At the species level, the Australian community possesses greater species richness in both *Sycoscapter* and *Sycophila*. In addition to these findings there may be an extra two wasp species in the Australian community whose status is ambiguous due either to their rarity or to the failure of COI gene amplification (Figs. 2, 3). Additionally, there are three individual wasps that display phylogenetic incongruence between their COI and ITS2 sequences.

### Comparing ecological guilds across regions

In China, *Acophila* and *Ormyrus* are additional large galler and large parasitoid genera not recorded in Australia. However, the inferred rarity of *Ormyrus* in China suggests that its apparent absence in Australia is unlikely to have any major ecological impact on community dynamics. In Australia, the addition of the small parasitoid, *Sycorycteridea*, is unlikely to alter community dynamics greatly, as it is closely allied to *Sycoscapter*, both taxonomically (both within tribe Sycoryctini [49]) and at a functional level (small parasitoids [50]). Furthermore, the Australian community has one or two extra species of small galler and one to three extra species of parasitoids (depending on the resolution of the exact number of species). Despite these differences, each community has all five fig wasp guilds (pollinators, small and large gallers, small and large parasitoids), as inferred from their taxonomic affiliations. While it would be of value to investigate patterns of relative wasp species abundances between these two communities, such analyses are beyond the scope of this work.

If *F. benjamina* presents the same niches across regions then community structure in China and Australia is predicted to be the same. However, a number of factors may prevent intercontinental ‘mirror-image’ communities. First, environmental conditions may differ between regions. However, there is no evidence from published *F. benjamina* fruit size data across regions to suggest any ecological differences that would present alternative niche opportunities typically associated with fig wasps [51–53]. Second, variation in regional species pools may have resulted in differential recruitment into communities after geographic separation [54]. For example, although *Ormyrus* is documented from Australian section Malvanthera *Ficus* species [55], phylogenetic constraints may prevent it colonising *F. benjamina,* which belongs to *Ficus* section Conosycea.

The extensive contiguous northern Asian landmass should theoretically host a greater wasp species pool compared to the northern tip of Australia due to its greater area (although it is thought that Australia was connected to New Guinea in the last 19–30 Ka [56]). In addition, Conosycea figs probably originated in Asia [57] and have greater extant diversity there than in Australia. Thus, the functional and compositional similarity between the communities suggest that any regional species pool differences have had little impact. Finally, differential extinction patterns may have occurred, causing some of the identified disparities in community composition.

### Historical divergence

Simultaneous divergence events were consistently supported over scenarios of multiple divergences according to guild (Table 3) and we suggest that most members of the extant Australian and Chinese communities are inherited from the same “ancestral community” that already had the major fig wasp guilds established. Differentiation between the extant communities has arisen primarily by allopatric divergence of these “inherited species”, alongside host range expansion by *F. benjamina*. Together, several lines of evidence support this scenario: (i) community-wide synchronised species divergences at appropriate timescales; (ii) conserved community composition involving persistent lineages; (iii) the posited low permeability to colonisation of fig microcosms; (iv) high levels of fig wasp host fidelity [58]. As such, these patterns suggest only a limited role for stochastic or phylogenetically constrained recruitment from regional species pools.

Such synchronised species divergences contrast with findings from Palearctic oak gall wasp communities, which are similarly structured around groups of specialised (albeit on *Quercus* (oak) sections) herbivorous insects and their parasitoid enemies [12]. Several non-exclusive factors could contribute to these differences: (i) the more stable climatic history of the (sub)tropical biome occupied by these figs when compared to temperate systems where demography has been shaped strongly by retreats into glacial refugia; (ii) lower host-plant specificity (generally host plant sections not species) for oak gall wasps than fig wasps; (iii) the mutualistic foundations of fig wasp communities that theoretically generate more tightly bound coevolutionary relationships. Thus, alongside past climatic considerations, relaxation of stringent interspecific relationships may allow a greater degree of flexibility among oak gall wasps. Furthermore, it is also possible that the large intercontinental distances between these Chinese and Australian communities have blurred these patterns, especially for the COI marker.

### Community composition and structure

No *F. benjamina* fig wasp species are shared between the Chinese and Australian communities, which are separated by > 5000 km. In contrast some African fig wasp communities showed low latitudinal turnover of species across > 4000 km [26, 28]. There are at least two possible, and not mutually exclusive, explanations for these differences: (i) the African study involves a contiguous landmass within one region, but our study compares two different biogeographic regions separated by archipelagic fragmentation, which probably represents a much stronger barrier to gene flow. (ii) The earlier African study [26] relied on morphological analyses of insects so may not have identified genetically distinct entities within morphospecies. Interestingly, a recent study of parasitoids in a different fig wasp community along the contiguous east coast of Australia [59] showed little turnover in morphospecies, but high turnover in cryptic species only recognised with molecular data.

Consideration of phylogenetic relationships suggests that these wasp communities may be shaped by ecological interactions between closely related species [60]. For seven species pairs in the three genera where intercontinental multispecies relationships are clearly resolved—*Walkerella*, *Philotrypesis* and *Sycophila*—each individual species’ closest relative is an intercontinental congener. This suggests that community functional structure has been consistent through space and time as congeneric lineages appear to have persisted rather than suffering lineage dropout and subsequent replacement by localised recruitment events or within community cladogenesis.

Among African section Galoglychia figs, community codivergence between several fig wasp lineages and their host fig species implicates cospeciation from an ancestral community [61]. This probably results from rigid coadaptive constraints determined by the fig syconia that select for high host fidelity [58]. For pollinating species these include optimal body size in order to enter the small regulatory entrance (ostiole) to the fig fruit and a coevolved ovipositor that allows exploitation of the fig flower gall sites. For parasitic species, ovipositor length and strength must be adapted to pierce latex-defended fig walls that vary between species in thickness and other traits [62]. These constraints further require wasps to evolve chemosensory recognition of species-specific fig volatiles to ensure they attack the correct host species. Thus, although we have shown compositional differences between the Chinese and Australian communities, the above factors probably ensure that the degree of differentiation is small compared to many more “open” invertebrate communities, e.g., disjunct bracken herbivore communities [11] or plant parasitic mites [54], that may recruit more from regional species pools.

BEAST divergence dating analyses between reciprocal congeneric Chinese and Australian inter-continental pairs generally suggest a split of around 4–16.5 Ma for these communities, with the higher values seen in *Philotrypesis*. Some caution is required with *Philotrypesis* as individual species have been found to utilise both *F. benjamina* and *F. microcarpa* in China [63]. Thus, host-switching has occurred in these figs and *Philotrypesis* lineage divergence may have occurred outside of *F. benjamina*. Moreover, relationships within *Philotrypesis* appear less clear (Figs. 2, 3) than for other taxa, which is consistent with data from Australasian (section Malvanthera) figs where *Philotrypesis* is thought to have entered the system relatively recently and shows little genetic divergence between otherwise clearly demarcated, morphologically distinct species [20, 58].

Otherwise, the deepest splits in reciprocal species are at ca. 15 Ma (*Sycobia*). However, the pollinating *Eupristina* wasps, which are the obligate insect component of the fig-wasp mutualism and are therefore expected to have undergone range expansions more or less contemporaneously with their host, appear to have split more recently, about 9 Ma. This paradox may be explained by the remarkable long-distance dispersal abilities of fig-pollinating wasps [64] that may augment species/population cohesion over greater distances than for the other wasp species [65] (but see also [66]) and thus retard lineage splitting. Alternatively, *Sycobia* could have undergone a genuine early divergence. It belongs to a subfamily of large galling wasps (Epichrysomallinae) that can often develop in unpollinated figs and thus be partly independent of the pollinators. Indeed, *F. microcarpa*, a close relative of *F. benjamina*, has been planted extensively outside its natural range and a recent study reports cases where large gallers were the only wasps present at a given site [27].

These estimated timescales are later than the hypothesised radiation of section Conosycea from around 35 Ma proposed by Xu et al. [30] and the collision of the Australian tectonic plate with the Ontong Java plateau around 23–26 Ma [67], and likely correspond to the subsequent Sunda-Sahul crossing of the Wallace line. Although, dating estimates may be seen to support a divergence model of host tracking (Fig. 4), with parasitoid species diverging after their galling hosts, the large 95% HPD error bars coupled with our PyMsBayes clustering analyses argue against such a conclusion.

The wide geographic range of *F. benjamina* and other section Conosycea figs makes their wasp communities ideal candidates for studies of beta diversity patterns and their underlying processes [e.g. 68]. The closed nature of fig microcosms means they are far less open to invasion than many other insect/plant systems. Thus, their communities are more likely to be organised by a mixture of both evolutionary history and ecological processes, rather than from stochastic incursions from other systems. Their ranges from Australia, across the island archipelagos of Southeast Asia, and into China and India would facilitate the study of a large number of distinct communities with shared evolutionary histories, particularly in an island biogeography framework [69].

## Conclusions

It is unknown whether the composition and structure of communities utilising the same ecological resource generally remain stable across geographic regions, nor whether species richness and functional diversity are a result of the ecological saturation of available niches. While our results do show some differences in taxonomic composition at the genus level, the overwhelming picture is of near ‘mirror-image’ communities composed of matching functional groups of wasps. Moreover, evidence that these communities have synchronously diversified across Australasia suggests that historic community range expansions were undertaken by a full complement of functional groups and that species interaction dynamics and community structure likely remained relatively stable over evolutionary time and across inter-continental geographic scales. Such mirrored patterns are most readily explained by ecological saturation dictating community composition although further work is required to test this idea. Additionally, further work examining variation in the underlying ecological networks of these systems across biogeographic regions would be of significant value.

## Additional files

**Additional file 1: Figure S1.** jMOTU outputs for COI datasets. Largest inflection points indicate barcoding gaps. Species richness estimates are: a) *Eupristina*—two species; b) *Walkerella*—five species; c) *Philotrypesis*—eight species; d) tribe Sycoryctini—six species; e) *Sycobia*—two (or possibly six) species; *Sycophila*—five species. **Table S1.** Chosen models of molecular evolution in BEAST dating.

**Additional file 2: Figure S2.** Bayesian consensus phylogeny for COI data. Phylogenetic tree. **Figure S3.** Bayesian consensus phylogeny for ITS data. Phylogenetic tree.
