# Supplementary material for: Conserved community structure and simultaneous divergence events in the fig wasps associated with Ficus benjamina in Australia and China
Source: BMC Ecol. 2018 Apr 3;18:13. doi: 10.1186/s12898-018-0167-y (PMC5883542; doi:10.1186/s12898-018-0167-y)
Supplement: Supplementary file 1 — Additional file 1: Figure S1. jMOTU outputs for COI datasets. Largest inflection points indicate barcoding gaps. Species richness estimates are: a) Eupristina—two species; b) Walkerella—five species; c) Philotrypesis—eight species; d) tribe Sycoryctini—six species; e) Sycobia—two (or possibly six) species; Sycophila—five species. Table S1. Chosen models of molecular evolution in BEAST dating. [file 12898_2018_167_MOESM1_ESM.docx]

Figure S1.


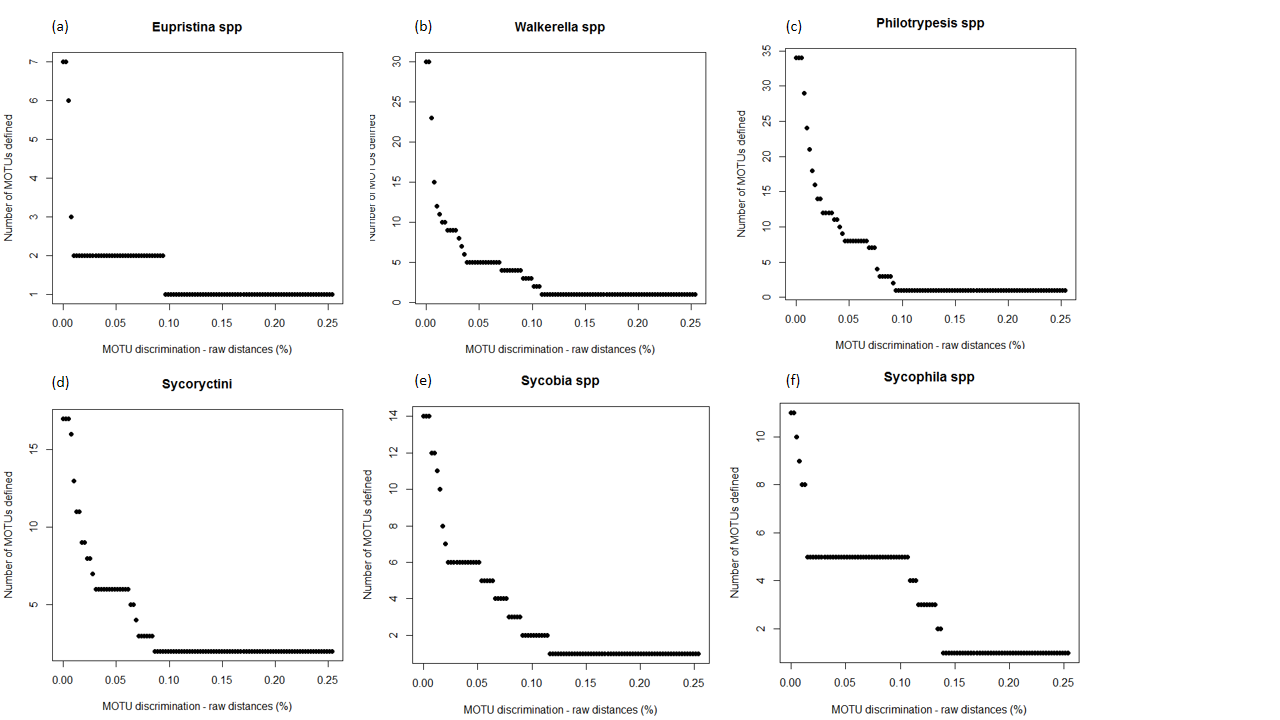


Table S1. Models of sequence evolution used in BEAST dating.

| **Taxon** | **Codon partitioning** | **Sequence Evolution** | **Molecular clock** | **Tree prior** |
| --- | --- | --- | --- | --- |
| *Walkerella* | All separate | HKY+I+G | Exponential relaxed | Constant coalescent |
| *Philotrypesis* | All separate | GTR+G | Exponential relaxed | Exponential coalescent |
| *Sycophila* | All separate | HKY+G | Exponential relaxed | Constant coalescent |
| Epichrysomallinae | All separate | HKY+G | Exponential relaxed | Constant coalescent |
| Sycoryctini | All separate | HKY+G | Strict | Constant coalescent |
