# Supplementary figures and images for: Conserved community structure and simultaneous divergence events in the fig wasps associated with Ficus benjamina in Australia and China
Source: BMC Ecol. 2018 Apr 3;18:13. doi: 10.1186/s12898-018-0167-y (PMC5883542; doi:10.1186/s12898-018-0167-y)

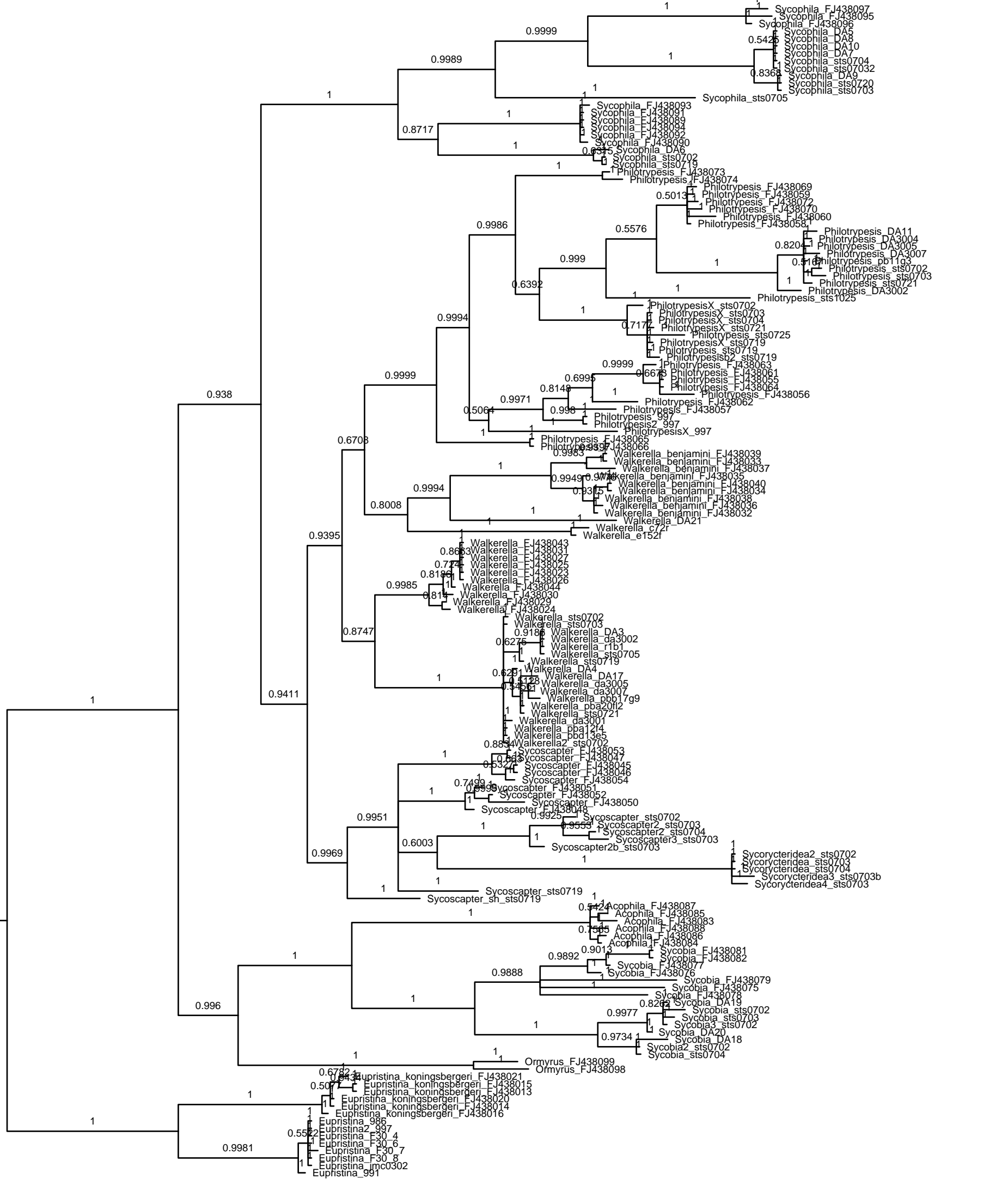

0.06

Supplement: Supplementary file 2 — Additional file 2: Figure S2. Bayesian consensus phylogeny for COI data. Phylogenetic tree. Figure S3. Bayesian consensus phylogeny for ITS data. Phylogenetic tree. [file 12898_2018_167_MOESM2_ESM.zip › SuppliInfo2/Figure S2.pdf]
