# Supplementary material for: Conserved community structure and simultaneous divergence events in the fig wasps associated with Ficus benjamina in Australia and China
Source: BMC Ecol. 2018 Apr 3;18:13. doi: 10.1186/s12898-018-0167-y (PMC5883542; doi:10.1186/s12898-018-0167-y)
Supplement: Supplementary file 2 — Additional file 2: Figure S2. Bayesian consensus phylogeny for COI data. Phylogenetic tree. Figure S3. Bayesian consensus phylogeny for ITS data. Phylogenetic tree. [file Figure S3.pdf › SuppliInfo2/Figure S3.pdf]

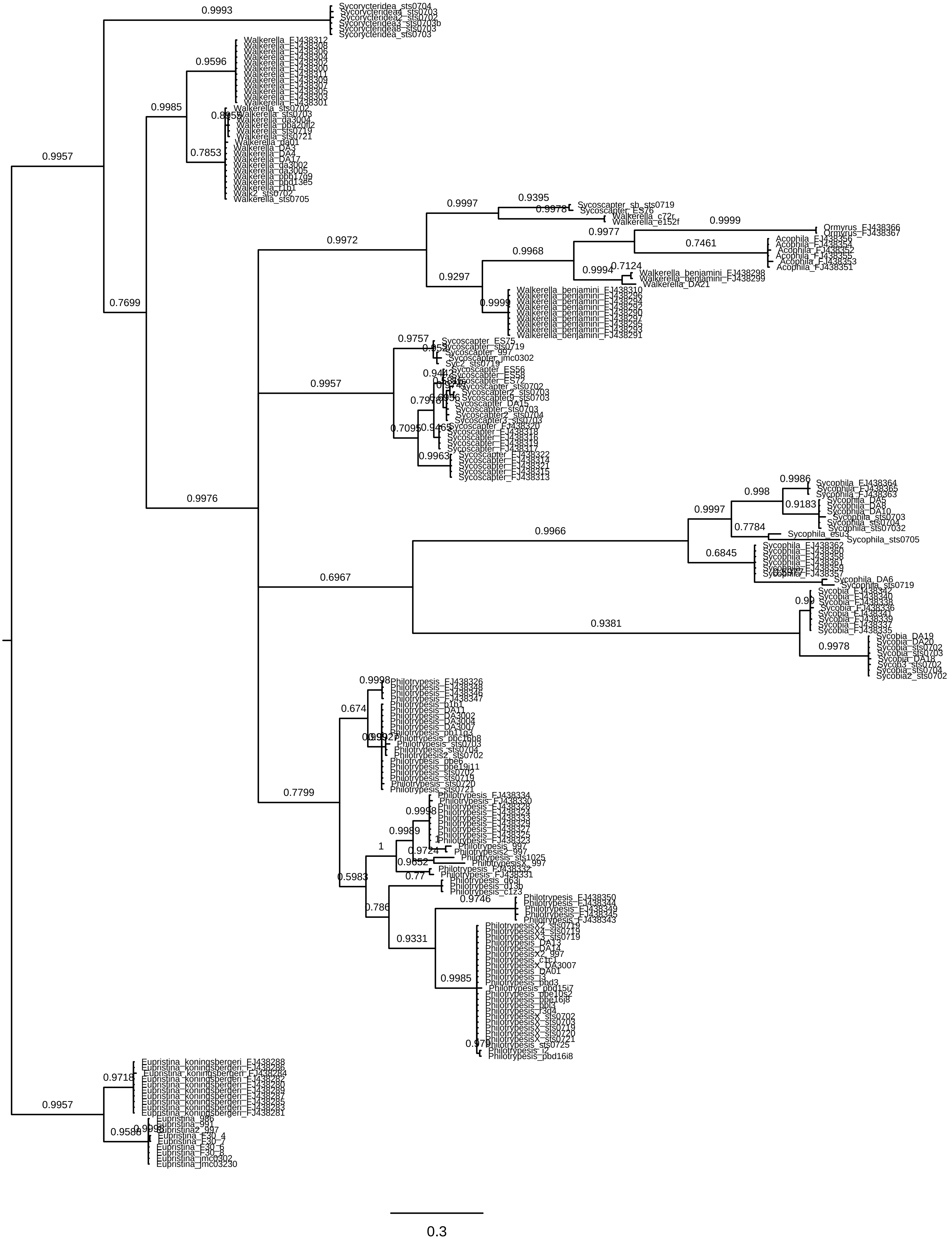

0.9993
Sycorycteridea_sts0704
Sycorycteridea4_sts0703
Sycorycteridea2_sts0702
Sycorycteridea3_sts0703b
Sycorycteridea8_sts0703
Sycorycteridea_sts0703
0.9957
0.9596
Walkerella_FJ438312
Walkerella_FJ438308
Walkerella_FJ438306
Walkerella_FJ438304
Walkerella_FJ438302
Walkerella_FJ438300
Walkerella_FJ438311
Walkerella_FJ438309
Walkerella_FJ438307
Walkerella_FJ438305
Walkerella_FJ438303
Walkerella_FJ438301
0.9985
Walkerella_sts0702
Walkerella_sts0703
Walkerella_da3004
Walkerella_pba20fl2
Walkerella_sts0719
Walkerella_sts0721
Walkerella_da01
0.7853
Walkerella_DA3
Walkerella_DA4
Walkerella_DA17
Walkerella_da3002
Walkerella_da3005
Walkerella_pbb17g9
Walkerella_pbd13e5
Walkerella_f1b1
Walk2_sts0702
Walkerella_sts0705
0.9997
0.9395
Sycoscapter_sb_sts0719
0.9978
Sycoscapter_ES76
Walkerella_c72r
Walkerella_e152f
0.9999
Ormyrus_FJ438366
Ormyrus_FJ438367
0.9972
0.9977
0.7461
Acophila_FJ438356
Acophila_FJ438354
Acophila_FJ438352
Acophila_FJ438355
Acophila_FJ438353
Acophila_FJ438351
0.9968
0.9994
0.7124
Walkerella_benjamini_FJ438298
Walkerella_benjamini_FJ438299
Walkerella_DA21
0.9297
0.7699
0.9999
Walkerella_benjamini_FJ438310
Walkerella_benjamini_FJ438296
Walkerella_benjamini_FJ438294
Walkerella_benjamini_FJ438292
Walkerella_benjamini_FJ438290
Walkerella_benjamini_FJ438297
Walkerella_benjamini_FJ438295
Walkerella_benjamini_FJ438293
Walkerella_benjamini_FJ438291
0.9757
Sycoscapter_ES75
Sycoscapter_sts0719
Sycoscapter_997
Sycoscapter_jmc0302
Syc2_sts0719
0.9957
Sycoscapter_ES56
Sycoscapter_ES58
Sycoscapter_ES72
Sycoscapter_sts0702
Sycoscapter2_sts0703
Sycoscapter5_sts0703
0.7978
Sycoscapter_DA15
Sycoscapter_sts0703
Sycoscapter2_sts0704
Sycoscapter3_sts0703
0.7095
Sycoscapter_FJ438320
Sycoscapter_FJ438318
Sycoscapter_FJ438316
Sycoscapter_FJ438319
Sycoscapter_FJ438317
0.9963
Sycoscapter_FJ438322
Sycoscapter_FJ438314
Sycoscapter_FJ438321
Sycoscapter_FJ438315
Sycoscapter_FJ438313
0.9976
0.9986
Sycophila_FJ438364
Sycophila_FJ438365
Sycophila_FJ438363
0.998
0.9183
Sycophila_DA5
Sycophila_DA8
Sycophila_DA10
Sycophila_sts0703
Sycophila_sts0704
Sycophila_sts07032
0.9997
0.7784
Sycophila_esu3
Sycophila_sts0705
0.9966
0.6845
Sycophila_FJ438362
Sycophila_FJ438360
Sycophila_FJ438358
Sycophila_FJ438361
Sycophila_FJ438359
Sycophila_FJ438357
Sycophila_DA6
Sycophila_sts0719
0.6967
Sycobia_FJ438342
Sycobia_FJ438340
Sycobia_FJ438338
Sycobia_FJ438336
Sycobia_FJ438341
Sycobia_FJ438339
Sycobia_FJ438337
Sycobia_FJ438335
0.9381
Sycobia_DA19
Sycobia_DA20
Sycobia_sts0702
Sycobia_sts0703
Sycobia_DA18
Sycob3_sts0702
Sycobia_sts0704
Sycobia2_sts0702
0.9978
0.9998
Philotrypesis_FJ438326
Philotrypesis_FJ438348
Philotrypesis_FJ438346
Philotrypesis_FJ438347
0.674
Philotrypesis_b1b1
Philotrypesis_DA11
Philotrypesis_DA3002
Philotrypesis_DA3004
Philotrypesis_DA3007
Philotrypesis_pb1103
Philotrypesis_pbc16h8
Philotrypesis_sts0703
Philotrypesis_sts0704
Philotrypesis2_sts0702
Philotrypesis_pbe6
Philotrypesis_pbe19j11
Philotrypesis_sts0702
Philotrypesis_sts0719
Philotrypesis_sts0720
Philotrypesis_sts0721
0.7799
Philotrypesis_FJ438334
Philotrypesis_FJ438330
0.9998
Philotrypesis_FJ438328
Philotrypesis_FJ438324
Philotrypesis_FJ438333
Philotrypesis_FJ438329
Philotrypesis_FJ438327
Philotrypesis_FJ438325
Philotrypesis_FJ438323
0.9989
Philotrypesis_997
Philotrypesis2_997
0.9724
Philotrypesis_sts1025
PhilotrypesisX_997
0.9652
Philotrypesis_FJ438332
Philotrypesis_FJ438331
0.5983
0.77
Philotrypesis_d63j
Philotrypesis_d13b
Philotrypesis_c1z3
0.9746
Philotrypesis_FJ438350
Philotrypesis_FJ438344
Philotrypesis_FJ438349
Philotrypesis_FJ438345
Philotrypesis_FJ438343
0.786
0.9331
PhilotrypesisX2_sts0719
PhilotrypesisX4_sts0719
PhilotrypesisX3_sts0719
Philotrypesis_DA13
Philotrypesis_DA14
PhilotrypesisX2_997
Philotrypesis_c1c1
PhilotrypesisX_DA3007
Philotrypesis_DA01
Philotrypesis_f3
Philotrypesis_pbd3
Philotrypesis_pbd15i7
Philotrypesis_pbe10s2
Philotrypesis_pbe16j8
Philotrypesis_pbl3
Philotrypesis_f3g4
PhilotrypesisX_sts0702
PhilotrypesisX_sts0703
PhilotrypesisX_sts0719
PhilotrypesisX_sts0720
PhilotrypesisX_sts0721
Philotrypesis_sts0725
Philotrypesis_f2
Philotrypesis_pbd16i8
0.9985
0.9718
Eupristina_koningsbergeri_FJ438288
Eupristina_koningsbergeri_FJ438286
Eupristina_koningsbergeri_FJ438284
Eupristina_koningsbergeri_FJ438282
Eupristina_koningsbergeri_FJ438280
Eupristina_koningsbergeri_FJ438289
Eupristina_koningsbergeri_FJ438287
Eupristina_koningsbergeri_FJ438285
Eupristina_koningsbergeri_FJ438283
Eupristina_koningsbergeri_FJ438281
0.9957
Eupristina_986
Eupristina_997
Eupristina2_997
Eupristina_F30_4
Eupristina_F30_7
Eupristina_F30_6
Eupristina_F30_8
Eupristina_jmc0302
Eupristina_jmc03230
0.9588
0.3
